# A Demonstration of the Curability of Pulmonary Consumption in All Its Stages

**Published:** 1843-06-24

**Authors:** 


### BIBLIOGRAPHICAL NOTICES.

*A Demonstration of the Curability of Pulmonary Consumption in all its stages. Comprising an inquiry into the nature, causes, symptoms, treatment, and prevention of Tuberculous Diseases in general.* By Wm. A. McDowell, M. D. Louisville, Ky., 1843. 8vo. pp. 269.

We cannot admit that Dr. McDowell's *demonstration* has brought conviction to our minds. He seems an honest, as well as ardent advocate of his system, but he has wofully neglected the proofs, in his attempt at proselytism. The cases which are recorded are few, and very meager in detail. There is no attempt at tabular or statistical evidence, and we are required to adopt implicitly the author's assertion. At the present day something more is required in medicine, and the profession demands facts upon which it can itself decide. Common salt—"the *salt of the earth, nature's remedy*"—would seem to be the mighty weapon by which the miracle announced in the title page is to be wrought. The relish for salt being instinctive in man as well as animals, those of the human race who are afflicted with a strumous diathesis particularly affect it. Tuberculous children who have not this saline craving rarely recover, according to our author; they prefer, clay, chalk, &c. The popular notion that indulging freely this brackish disposition, will "dry up the blood," is ably combatted, and a case cited as proof to the contrary.

"Muriate of soda, as a medicine, is a perfect proteus," (p. 141,) and we accordingly find that it covers the whole materia medica, and that we poor mortals are daily dosing ourselves, not only with one powerful medicine, but actually swallowing the whole pharmacopœia at each dinner—an astringent, a purgative, a tonic, an emetic, a stimulant, an anti-irritant, (?) a styptic, a rubefacient, &c., and besides all these eminently useful properties—combined in one compound, and acting instinctively, when necessary, in each case,—"that it is the cause of fatal hæmorrhage in scurvy," p. 151. The very inhalation of it would, in some cases, seem to be sufficient, p. 247.

In spite of its unprepossessing title, and the signal failure to fulfil the promise made in it, there is much in the book before us to redeem it, and to show that its author is a man of no limited experience, and endowed with discretion and judgment.

